# Recent Developments in Solid Lipid Microparticles for Food Ingredients Delivery

**DOI:** 10.3390/foods10020400

**Published:** 2021-02-11

**Authors:** Victoria Nahum, Abraham J. Domb

**Affiliations:** Faculty of Medicine, Institute of Drug Research, School of Pharmacy, The Hebrew University of Jerusalem, Jerusalem 91120, Israel; avid@ekmd.huji.ac.il

**Keywords:** solid lipid microparticles, lipid carriers, food bioactives, microencapsulation, bioavailability

## Abstract

Health food has become a prominent force in the market place, influencing many food industries to focus on numerous bioactive compounds to reap benefits from its properties. Use of these compounds in food matrices has several limitations. Most of the food bio-additives are sensitive compounds that may quickly decompose in both food and within the gastrointestinal tract. Since most of these bioactives are highly or partially lipophilic molecules, they possess very low water solubility and insufficient dispersibility, leading to poor bioavailability. Thus, various methods of microencapsulation of large number of food bioactives have been studied. For encapsulation of hydrophobic compounds several lipid carriers and lipid platforms have been studied, including emulsions, microemulsions, micelles, liposomes, and lipid nano- and microparticles. Solid lipid particles (SLP) are a promising delivery system, can both deliver bioactive compounds, reduce their degradation, and permit slow and sustained release. Solid lipid particles have important advantages compared to other polymer carriers in light of their simple production technology, including scale up ability, higher loading capacity, extremely high biocompatibility, and usually low cost. This delivery system provides improved stability, solubility in various matrixes, bioavailability, and targeting properties. This article reviews recent studies on microencapsulation of selected bioactive food ingredients in solid lipid-based carriers from a point of view of production methods, characteristics of obtained particles, loading capability, stability, and release profile.

## 1. Introduction

### 1.1. Food Additives

Food additives are substances incorporated in foods to improve production, processing, storage or packaging, to increase or maintain nutritional values, to maintain or improve safety, freshness, and taste, as well as texture and appearance [1]. Production and use of new additives decidedly multiplied during the 20th century because of the increased number of industrial foods. Almost all newly invented products, such as snacks, low-calorie, and ready-to-eat foods could not have been possible without food additives. There is also a growing demand for the incorporation of active nutritional ingredients in food for health reasons, as depicted in following table (Table 1).

Food additives can be categorized [3] as preservatives, nutritional additives, flavoring agents, coloring agents, etc. Some can belong to more than one category. *Food preservatives* are compounds with antioxidant or antimicrobial properties. Antioxidants supplements allow to delay or prevent decomposition of foods due to oxidation during industrial processing, packaging, or storing. Compounds for this purpose include, among others, Ascorbic acid (AA) as an oxygen scavenger, and Butylated hydroxyanisole (BHA) or Butylated hydroxytoluene (BHT) as free radical scavengers. Antimicrobial agents such as benzoic acid, natamycin, nitrates, sorbic acid, parabens, and others inhibit growth of bacteria or other microorganisms in foods. *Nutritional additives* are usually added to enrich various foods with dietary supplements [4]. For example, vitamins are added to many foods to rise their nutritional value. For example, Vitamin A and D supplements can be added to dairy and cereal products. Vitamins B are added to flour, pasta, cereals, and baked goods. Vitamin C is added to fruit beverages, cereals, and dairy products. Probiotics, as well, are an example of an additive commonly added to dairy products to enrich the gastrointestinal flora with essential microbes. Fatty acids, minerals, and dietary fibers are other examples of nutritional additives used to enrich food products. Various texturizing agents are added during food production to supply the desired consistency of a product. Our perception of product quality is often heavily influenced by its color. Therefore, *colorants* are added to create a standard product with an attractive color. These coloring agents can be divided into two categories: natural or synthetic. Natural colorants can originate from plants (e.g., strawberry (red), grape (blue), paprika (red/orange)), from animal or mineral sources, while artificial food colorants are usually petroleum-based chemical compounds (e.g., Brilliant Blue FCF, Indigotine, Fast Green FCF, Allura Red AC, Erythrosine, and Tartrazine). The largest group of food additives, with more than 1200 different compounds that can create flavor or replenish flavors lost or diminished in processing is *Flavoring agents*. They can be only one chemical or a mixture of several chemicals drawn from natural or synthetic sources.

### 1.2. Encapsulation of Phytochemicals and Food Additives

**Figure 1:**
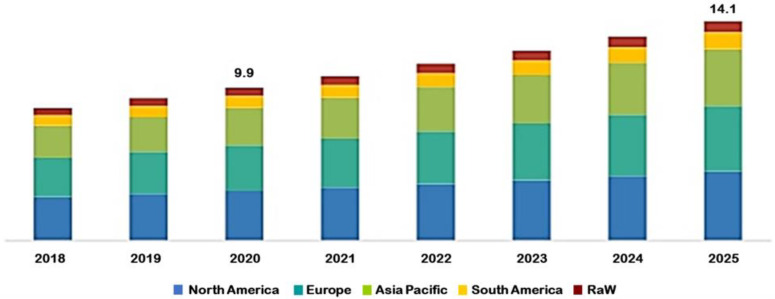
Projection of food encapsulation market.

For health concerns the food industry avails itself of increasingly more natural substances as food bio-additives. Due to processing and gastrointestinal conditions, there is a growing demand to initiate new processes and technologies suitable for their protection [5], since these materials are commonly recognized as highly sensitive to the environment, like heat, light, oxygen, and moisture. One proposed tool that can ensure improved bioavailability, stability, and delivery of these materials is microencapsulation [6], that can potentially prevent interaction with the food matrix and convert the applied ingredients into the easily handled, free-flowing, and dispersible powders. Taste masking is another aim of encapsulation. Essential fatty acids, for example, are highly valuable for health, however they have an unpleasant taste that can become a negative factor when overgoing oxidation process. This problem can be avoided by encapsulation in suitable vehicles. Pre-cooked food, for example, represents a large part of our consumption. Recomposed powders, long-term storage period, and the need for innovation have deeply changed the management of the products [7]. As mentioned previously, microencapsulation offers sustained and controlled release that can be prompted by different factors such as dissolution, temperature, pressure, pH, and enzymes, depending on wall material [8]. The global food encapsulation market was estimated to account for US $9.9 billion in 2020 and is projected to surpass US $14 billion by 2025, (Figure 1) [9]. This market is mainly driven by the rising request for encapsulated flavor and colorants. High demand for fortified and functional foods among consumers has caused a rise in demand for food additive encapsulation. Manufactures, therefore, have been adopting this technology at a high rate.

### 1.3. Examples of Microencapsulation of Bioactives in Foods

Microencapsulation is a process in which small particles or droplets of a core material are coated or embedded into a homogeneous or heterogeneous matrix to create small capsules. Microcapsules are extremely small (1–1000 µm) and can have numerous morphologies. Food microcapsules, in particular, are small particles containing an active food additive (core) surrounded by a coating or shell. The shell material can comprise a variety of food grade polymers, carbohydrates, fats, and waxes that function as a barrier between the bioactives and the surrounding environment. Encapsulation of flavors, lipids, and carotenoids among other ingredients had recently attracted much attention by food industry [10]. l-Menthol, for example, has been encapsulated in a carrier mixture of maltodextrin and gum Arabic. Flavors such as oregano, citronella, and marjoram have been encapsulated using skimmed milk powder and whey protein concentrate [11]. Flavor-loaded microcapsules can release the active material at a perfect time to produce the maximum effect for the consumer. Microencapsulated fish oil (an oil rich in omega-3 polyunsaturated fatty acids) powder with hidden taste and prolonged shelf life has been successfully manufactured and introduced into numerous food products. Lycopene, a molecule believed to decrease the risk of prostate cancer, has been successfully microencapsulated using a carrier consisting of gelatin and sucrose [12]. Another use of microencapsulation is to reduce degradation of carotenoids in foods, thereby preventing decreased product quality in nutritional properties and coloration [11]. Specific applications of microencapsulation are [13]: baby foods, baked products, chewing gum, flavored waters, leavening agents, nutrition bars, and yogurt. Taste-masked microcapsules containing caffeine for food and sports nutrition industries have been produced for gels or chews to avoid typical unpleasant taste [14]. Microencapsulation has also been used to improve water spreading and bioavailability of hydrophobic materials, such as coenzyme Q10 or carotenoids [15,16].

### 1.4. Microencapsulation of Phytochemicals and Food Additives Using Polysaccharides and Proteins

Guidelines for food additives are more severe than for pharmaceuticals. Many carrier materials are not “generally recognized as safe” (GRAS) and have not been certified for food applications [6]. Therefore, natural materials were used mostly for encapsulation in the food industry. Polysaccharides are the most broadly used natural polymers in food applications. These natural carbohydrate polymers originate from different sources, e.g., plants, animals, algae, and microbes. Starch is one of the most abundant polysaccharides in plants. Frequently used starches and its derivatives include amylose, amylopectin, dextrins, cyclodextrines, maltodextrins, syrups, polydextrose, and cellulose. Another group of natural polysaccharides enjoying wide food applications is gums, due to their exceptional functionalities, non-toxicity, biodegradability, biocompatibility, and safety for human nutrition. Gums possess an excellent ability to incorporate flavors, nutraceutical compounds, phenolic compounds, and antioxidants. Other polysaccharides utilized for encapsulation are carrageenans and alginates (from marine sources), dextran, chitosan, xanthan, and gellan (from microbial and animal sources). Proteins are also suitable for encapsulation since they are GRAS materials as well as they possess high nutritional values [17]. Proteins are found to be an effective vehicle for bioactive compounds, fats, oils, fatty acids, and flavors The most frequently used animal proteins in encapsulation are casein, gelatin, collagen, whey protein, while plant-derived proteins are soy protein, zein, and gliadin.

Although numerous reviews have been published concerning microencapsulation of food ingredients, we have tried to update recent developments in the *solid lipid encapsulation* of food bio-additives. The most recent (the last several years) research scientific original articles that develop food bioactives-loaded solid lipid microparticles are reviewed, emphasizing materials used, methods of particle preparation, particles characterization, and various examples of its applications in food industry.

## 2. Solid Lipid Encapsulation

Solid lipid micro- and nanoparticles, also called Lipospheres, are drug-loaded solid lipid spheres decorated with phospholipids and surfactants, used to deliver active agents [18,19,20,21]. Lipospheres are prepared by a melt process in which the lipid and the drug are melted and mixed with a hot buffer containing the phospholipids and surfactants, then homogenized to create a uniform emulsion. Upon rapid cooling a dispersion is formed. Alternatively, the drug, lipid, phospholipid, and surfactants can be dissolved in an organic solvent and evaporated in a large flak to form a thin film. Upon addition of a warm buffer and homogenization, a lipid suspension is obtained. Lipospheres have been prepared for extended delivery of local anesthetics, vaccines, antibiotics, insect repellent, cosmetic agents, etc. [22]. An in-situ formulation called Pro-Nanodispersion Lipospheres (PNL) spontaneously forms nanoparticles upon addition to aqueous media. PNL are designed to improve the oral bioavailability of cyclosporin and cannabinoids [21,22,23,24,25,26,27]. Upon contact with GI (gastrointestinal) fluids, the oily formulation loaded into soft gelatin capsules immediately forms lipid nanoparticles of 30 nm to significantly improve the oral bioavailability of Cyslosporin [21]. Similar formulations to improve oral delivery of cannabinoids are under clinical development [23,24,25,26,27]. Lipospheres, PNL, or Solid Lipid Nanoparticles (SLN) have potential applications in the delivery of water insoluble active agents by different routes of administration for improved bioavailability. Many bioactive ingredients, nutrients, and other food additives, including fatty acids, vitamins, polyphenols, aromas, and preservatives are highly hydrophobic materials. Absorption into the small intestine increases when an edible lipid is added, thereby promoting solubilization and transportation of lipophilic agents [28]. For these reasons micro- and nanoparticles produced from different lipid systems as transporters have been studied extensively. The presence of lipids can instantaneously increase the dispersibility of hydrophobic additives leading to improved absorption, and thus to increased bioavailability. The difference between solid lipid particles and oil-in-water (o/w) emulsions is that their lipid carrier is composed of a solid lipid (solid at room temperature) [29]. The added active materials (core) remain incorporated inside a solid lipid phase, usually stabilized by surfactant molecules [30]. The solid state of Solid Lipid Microparticles (SLM) provides clear advantages over the liquid state, encouraging increasing interest in their development. Lipid microparticles can be an advantageous alternative to polymeric particles in light of several visible advantages of this delivering system. Amongst them are the facility of large-scale production, relatively low cost, and low toxicity. In addition to solubilization and better dispersion of the active ingredient, SLM can provide extended release of active agent, to mask the undesirable taste and protect against degradation inside the GI tract [31]. Many lipid materials are suitable for food applications, including fatty acids and fatty alcohols, waxes, and triglycerides. Usually, the solid lipids used in the microencapsulation process have a melting point from 50° to 85 °C [32]. Lipid transporters have exceptional properties that widely used in emulsification, film formation, and encapsulation of active compounds thereby providing many potential applications in the food industry [33]. Polar lipids, such as biocompatible phospholipids, are good surface-active compounds suitable for the stabilization, protection, and controlled release of active compounds [34]. Therefore, lipid-based encapsulation techniques can certainly offer a number of important benefits as a shield system for sensitive food bioactives.

## 3. Ingredients Used for the Preparation of SLM

Encapsulating materials employed for solid lipid particles preparation are selected for their biocompatibility, availability, and price. A successful application of the microencapsulation technique depends primarily on the correct choice of solid lipids with desired physicochemical and rheological properties for each specific bioactive. Many different materials can be employed in this process; however, they must be biodegradable and approved as GRAS for food applications [35]. They must also preserve bioactive compounds from decomposition during industrial processing and support conditions of storage. They should not interact chemically with other incorporated active agents. Several solid lipids have been used extensively. They can be divided into several categories, including waxes, vegetable oils, fatty acids, fatty alcohols, and triglycerides. Table 2 contains examples of materials belonging to each group.

It is very important to choose the most appropriate encapsulating carrier or mixture of carriers to achieve higher retention of active material inside the particles, since composition can contribute to overall stability. For example, probiotics can be protected during the digestion process using *vegetable fat* as the encapsulating carrier, thereby ensuring functionality [36]. *Vegetable fat* has also been used to encapsulate Vitamin D3, since it has a suitable melting point (above room temperature, but not too high), which is important in avoiding decomposition of vitamin D3 during the particle formation process. Vegetable fat is also edible and commercially available. It is widely used to prepare ice cream, bread, and other food products [37]. *Palm stearin* lipid has been used for SLM production due to its special triglyceride composition that promotes a disorganized, less crystalline structure to prevent rejection of the encapsulated bioactives during the solidification process [38,39,40]. Lipids with relatively low melting points have been studied extensively in microencapsulation; but the use of materials with high melting points is not as common. The use of high melting point lipids can be interesting for possibly increased stability compared to low melting point lipids [41]. *Carnauba wax*, obtained from the Brazilian carnauba palm tree, is a solid lipid material consisting of a mixture of fatty esters, fatty alcohols, and fatty acids, with a high melting temperature (82 °C–86 °C). For example, Quercetin, a plant-derived flavonoid, was successfully microencapsulated by using carnauba wax for taste-masking [42]. Lipid mixtures were used for encapsulation as well. Thus, mixtures composed of *palmitic acid* with *oleic acid* or *palm fat* were used as carriers for encapsulation of Ginger oleoresin, a flavor ingredient also known as an antimicrobial and antioxidant agent [43]. Ginger oleoresin has been encapsulated using a lipid matrix containing a mixture of solid and liquid lipids. In this study, *stearic acid* was used as a solid lipid and *oleic acid* as a liquid lipid [44]. Solid lipid microparticles containing Ascorbic acid (Vitamin C) were synthesized, as well, using mixtures of *Lauric acid* (LA)/*Oleic acid* (OA) at different ratios [45]. For the encapsulation of fish oil, *fully hydrogenated soybean oil* was chosen as a solid lipid, since it is inexpensive material (widely used for the preparation of candles) and stable against oxidation [46]. Curcumin was encapsulated in solid lipid microparticles formed from a lipid mixture containing *Babacu oil* and *Tristearin*, followed by incorporation into low-sugar mango jam [47].

The selection of surfactants to produce solid lipid microparticles is an important factor when considering incorporation into foodstuffs, since many of the compounds usually useful for particle stabilization are not of food grade. Among the GRAS surfactants, *protein concentrates* and *isolates* are used for microencapsulation. SLM containing β-carotene for incorporation into yogurts and vanilla ice creams was stabilized with *hydrolyzed soy protein isolate* [39,48]. Solid lipid microparticles can be produced from babacu oil as a lipid carrier, and denatured *whey protein isolate* can be used as a stabilizer to inhibit quercetin degradation [29]. Lacprodan 80 *whey protein concentrate* (WPC) has been used to produce microparticles containing a proanthocyanidin-rich cinnamon extract (PRCE) [49]. Another emulsifier used for the SLM preparation is *plant* or *animal lecithin*. *Soy lecithin* has been incorporated into the SLM containing vitamin B12 (Cyanocobalamin) [50] and vitamin D3 [37]. Tween 80 and Span 60 were used as surfactants for the encapsulation of curcumin [47] as well as for the formation of Chia oil-loaded microparticles [51].

## 4. Methods for the Solid Lipid Microparticles Preparation

Different methods of microencapsulation can be used for various materials. The choice of the most suitable method depends on the type of active ingredient, its special function, and the desired application. There is no single method that can be universally applicable for the encapsulation of every food additive. Several techniques have been cited in the literature for SLM preparation [13]. Each method has its own strengths and weaknesses in encapsulation, cost, ease of use, as well as morphology and size of formed particles. To achieve the maximum quality of the loaded microparticles, the encapsulation method must enable the formation of particles with a high loading capacity, high encapsulation efficiency, stability of encapsulate system during industrial processing and during storage, essential release features, and bioavailability of the active agent [13]. Encapsulation is a rapidly developing technology with strong potential in the food industry. Among the many encapsulation techniques, several can be employed for the preparation of solid lipid microparticles. These include Spray Drying, Spray Chilling/Cooling, Extrusion, and Hot Homogenization. Table 3 summarizes the main principles of each method.

### 4.1. Spray Drying Technique

**Figure 2:**
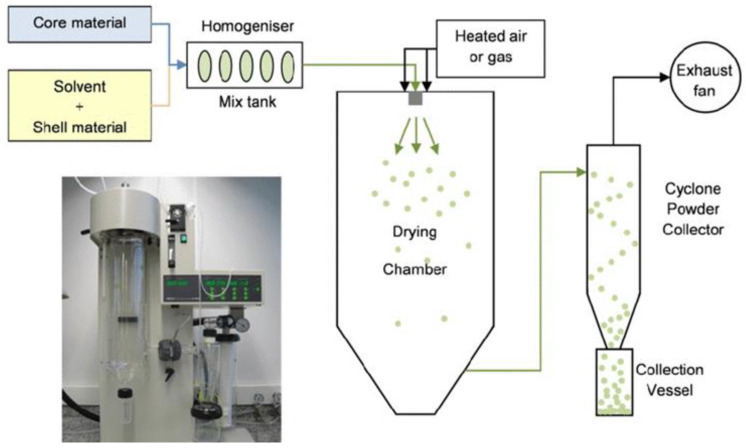
Spray drying apparatus.

Spray drying (SD) is the oldest and most widely used microencapsulation method in the food industry [52]. SD is flexible and cost-effective, allowing continuous production. It can be easily industrialized in terms of equipment and materials that have a low cost compared to other techniques. SD produces high quality microparticles by atomizing a hot liquid oil mixture or emulsion through a nozzle into a hot gas chamber, thus producing a particles powder. The spray-drying process has three steps: (i) Preparation of the dispersion or emulsion containing the active material, (ii) homogenization of the dispersion, and (iii) atomization of the resultant mixture into the drying chamber (Figure 2 [53]). Despite the high temperatures generally used in this process (~180 °C), the core temperature remains below 40 °C because of the short time exposition and rapid evaporation of water. SD usually produces hollow, low-density particles with an irregular geometry. The size of the obtained microparticles can vary from 10–50 µm at the fine end to 2–3 mm at the large end (Table 4), depending upon concentration of the starting solution and operating conditions [54].

SD can be used to encapsulate different food ingredients, e.g., omega-3 oils, enzymes, bioactive peptides, molecules, probiotics, phenolic compound, antioxidants, antimicrobial agents, vitamins, natural food colorants, flavors, and essential oils [55]. A caramel flavor was successfully microencapsulated in a coffee containing milk beverage using spray drying technique [56]. Microencapsulation of flavor and the addition of the microcapsules to coffee beverages was efficient to prolong flavor retention and improve beverage quality. In a different study, water-dispersible microcapsules containing natural colors such as Anthocyanin and Norbixin were encapsulated into a two-layer shell using a SD technique [57]. An inner, solid lipid layer consisted of glycerol mono-oleate (GMO) and soy lecithin, and an outer, water-dispersible system consisted of maltodextrin and poloxamer 338. Anthocyanin- and Norbixin-loaded microcapsules were designed and produced to protect the heat-sensitive colorants from thermal degradation during industrial processing. SD is one of the most commonly used commercial methods for microencapsulation of omega-3 fatty acids as well [54]. Although spray drying is a common technique, relatively high temperatures used in the drying chamber can harm natural sensitive compounds such as vitamins, lycopene, β-carotene, colorants, and flavors [58]. In addition, since this technology uses air at very high temperatures, particles with a porous structure can be produced, leading to decreased stability and reduced shelf-life [54].

### 4.2. Spray Chilling Technique

**Figure 3:**
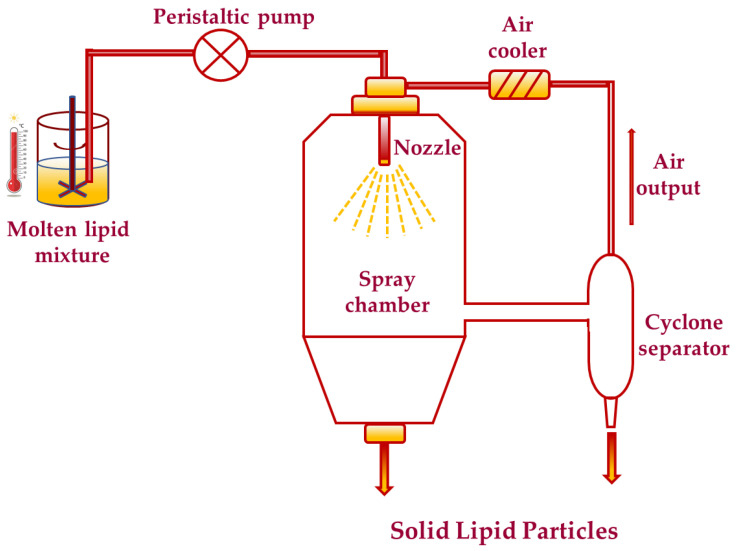
Schematic representation of standard spray congealing setup.

In contrast, the spray chilling/coagulating/cooling (SC) method is based on the atomization of a hot mixture of the active material and lipid carrier in a cold chamber, in which small hot droplets solidify in contact with the cool air to form solid lipid microparticles (SLM) [59]. This method does not require the use of high temperatures, water, or organic solvents during the production, making it an environmentally friendly process [60]. In addition, in this method the solid lipid microparticles (SLM) are produced in milder temperatures, leading to a reduction in the degradation of sensitive compounds. The microparticles produced by spray chilling technique are dense and display a spherical shape (Table 4), with their active ingredients distributed homogenously throughout the volume of the particle [61]. According to the thermal stability of the active agent, a suitable lipid carrier may be chosen, thereby enabling the application of this technique to encapsulate thermolabile materials [62]. Since this technology is fat based, lipid carriers such as waxes, oils, fatty acids, pure triglycerides, or blends of these materials can be used [63]. The most frequently used lipid systems in SC method are fully hydrogenated fats of plant origin (hard fats) with melting points ranging from 36° to 75 °C. The general scheme of spray-chilling process is depicted in Figure 3 [64].

The spray chilling procedure consists of an initial heat-melting of the lipid system at 10 °C above its melting point. The molten suspension is then transported into a feeding reservoir placed above the spray-congealing nozzle, pre-heated to 10 °C above the melting temperature of the mixture. Atomization of the molten suspension is then achieved using a wide pneumatic nozzle. The atomized molten droplets solidify in a cooling chamber at room temperature to form spherical microparticles [32]. The efficiency of the process is one of the principal advantages related to this encapsulation technique. SC is a promising technique to produce solid lipid microparticles loaded with various heat-sensitive bio-additives. Thus, microparticles containing Ascorbic acid (AA, Vitamin C) are successfully produced by spray chilling using stearic acid and hydrogenated vegetable fat as wall materials [65]. The obtained microparticles displayed characteristic spherical morphology, high polydispersity, and high encapsulation efficiency (>97%) [59]. It was also possible to microencapsulate Ascorbic acid by spray chilling method using mixtures of two fatty acids, lauric acid and oleic acid, as a carrier lipid system. These mixtures were an exceptional lipid medium to control the release of ascorbic acid, showing potential application in food industry [65]. The same SC technique was used for the formation of solid lipid microparticles loaded with Ginger Oleoresin (GO); a flavor ingredient frequently used in foods. Lipid mixtures consist from palmitic acid with oleic acid or palm fat were successfully used as carriers, and as a result, high preservation of the volatile compounds of Ginger Oleoresin was accomplished [43,44]. This technique is also widely used in the microencapsulation of Essential Oils (EO) [66]. Hydrogenated vegetable oil or a wax were used as a molten carrier, creating almost perfectly spherical microparticles with a narrow size distribution. In another work, researchers evaluated the application of spray chilling technique to produce solid lipid microparticles containing Gallic acid as a model phenolic compound, using blends of soybean oil (SO) with fully hydrogenated soybean oil (FHSO) as lipid wall materials [62]. Probiotics are another active ingredient known for being unstable at elevated temperatures, therefore, using spray chilling process microparticles can be obtained with a survive of larger number of microbes [67]. In a recent study, probiotic microorganisms, Lactobacillus acidophilus (LA) and Bifidobacterium animalis subsp. Lactis (BL), have been encapsulated in solid lipid microparticles consisting from vegetable fat as a lipid carrier with a melting point of 51 °C. Results showed that spray chilling technique created a dry powder composed of smooth and continuous spheres and the microencapsulated probiotics presented viability at least of 90 days in savory cereal bars [67].

### 4.3. High-Shear Homogenization

**Figure 4:**
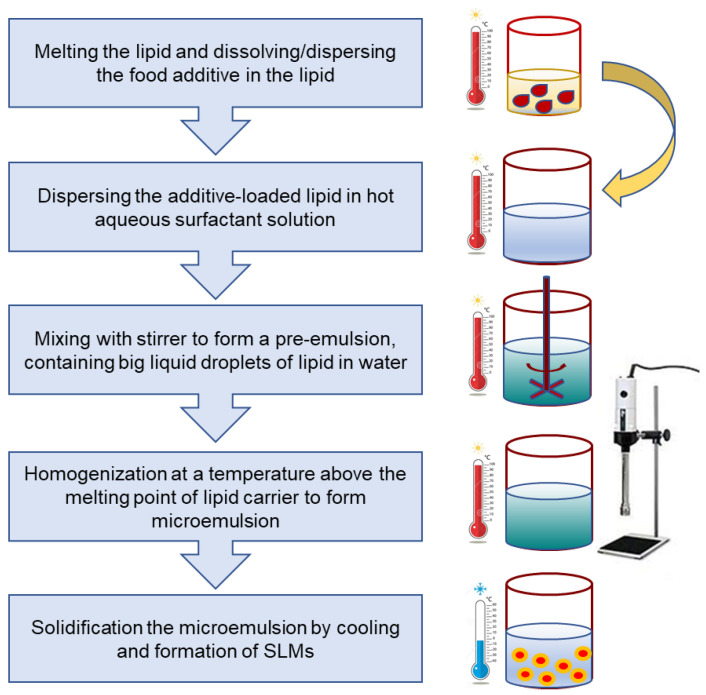
High-shear homogenization method.

High-shear homogenization is a simple method in which at a first step lipid is melted above its melting point, followed by addition of poorly water-soluble active agent. At the same time, an aqueous emulsifier solution is heated at the same temperature. In the second step, both materials are mixed and stirred at high-speed using high-shear homogenizer with continuing heating. Solid lipid particles are formed upon cooling of the molten mixture (Figure 4). This technique can be used for hydrophilic (cold homogenization) and lipophilic (hot homogenization) core molecules. A lesser quantity of emulsifier is required in this process in comparison to the microemulsion technique. Another advantage of high-shear homogenization technique is that lab-scale and large-scale production can also be readily accomplished [68].

The hot homogenization technique has been used for production of solid lipid microparticles containing tristearin and babacu oil as the carrier and denatured whey protein isolate (WPI) as a surfactant [29]. The research evaluated the ability of proposed lipid system to protect Quercetin, a flavonoid known for antioxidant and anti-inflammatory properties against degradation. The solid lipid microparticles were produced by melting the lipid phase at 70 °C and then adding the Quercetin. The hot WPI dispersion solution was mixed with the melted lipid followed by ultra-agitation at 12,000 rpm for 5 min. Results showed that the formed microparticles efficiently protected the Quercetin from oxidation, and 85% of the initial amount of the flavonoid was remained after 30 days. A high-shear homogenization method was also used to produce β-carotene-loaded solid lipid microparticles [39,69]. These SLM were prepared by dispersing the aqueous phase (containing hydrolysed soy protein isolate, HSPI) in the palm stearin with ultra-agitation at 15,000 rpm. The same technique was implemented for encapsulation of essential fatty acids. Hot homogenization was a suitable technique in view of the natural similarity and compatibility between polyunsaturated fatty acids (PUFA) and the solid lipid carrier. Chia oil-loaded solid lipid microparticles were obtained by hot homogenization, using stearic acid as a lipid carrier and Tween 80 as a surfactant at 75 °C [51]. Production of Curcumin-loaded solid lipid microparticles was achieved by hot homogenization, as well, using a mixture of tristearin and babacu oil as a lipid carrier and Span 80 as surfactant [47]. In another study, Curcumin-loaded SLM were produced using palm stearin as the lipid phase, and Tween 80 with Span 80 as hydrophilic and lipophilic surfactants at 80 °C, utilizing a rotor-stator device at 18,000 rpm [70]. Natural colorants were encapsulated in Glycerol mono-oleate (GMO) as a lipid carrier and soy lecithin as an emulsifier with a high-shear homogenizer at 15,000 rpm [57].

### 4.4. Melt Extrusion Technique

**Figure 5:**
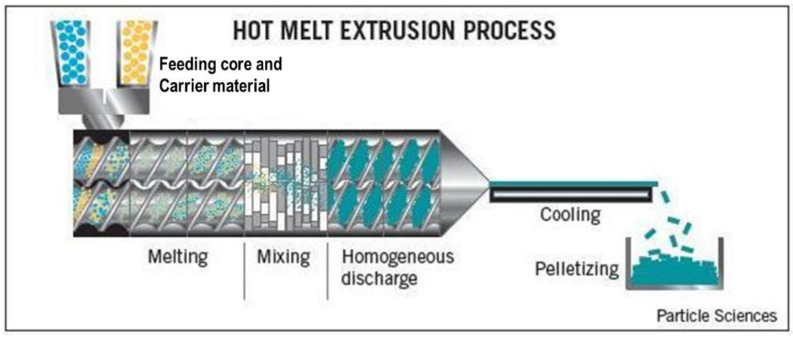
Hot-melt extrusion apparatus.

The earliest pioneering work using extrusion for encapsulation can be dated to the late 1950s in the flavor industry, known as melt injection [71]. Commercial extrusion technique for the microencapsulation of additives in food industry, however, is a relatively less used process. Extrusion is a procedure that enables formation of product with uniform shape and density from a raw material by forcing it through a die under controlled temperature, product flow, and pressure conditions. In a melt extrusion process the matrix components are melted, followed by mixing them with active compounds, thus allowing a smooth lipid mixture to be obtained. The technology is well industry-compatible, whose flexible configuration allows melting, addition, mixing, and cooling of the mixture in a continuous way. The melt extrusion technique includes the following stages: (1) melting of the carrier material inside a twin-screw extruder, (2) direct addition of an active compound, (3) dispersion of the active compound into the coating material, and (4) cooling and shaping of the extrudate, thus producing particles of 0.3–5 mm (Table 4). Hot-melt extrusion offers many advantages over other methods, for example simplicity, continuous operation, and high throughput [42]. During extrusion, active materials are mixed with molten excipients, producing homogenous matrix, following by cooling and formation of desired particles forms, like granules, sheets, or strands (Figure 5 [72]). This technology can be used to produce high-density encapsulated products. Melt extrusion promises encapsulation from the economic and environmental perspectives, since it is a one pot process (formation of the wall material, dispersion of the active principle, and forming of the encapsulated material), without use of any organic solvent as well as reduced energy and water consumption.

Hot-melt extrusion technique was successfully used for preparation of powdered Quercetin-loaded solid lipid microparticles composed from carnauba wax, shellac, and zein [42]. Physical mixtures were manually fed into a preheated twin-screw melt extruder. The extrusion was conducted continuously at a screw rotation speed of 100 rpm without use of a die. The resulting powders showed meaningfully reduced dissolution in the simulated salivary medium (pH 6.8), which can indicate that achieved efficiency of taste-masking was good. This process is especially suited for encapsulation of highly sensitive citrus flavors as well [71]. When rapidly dried, the extruder forms an amorphous, glassy mass that completely encloses flavor droplets, forming small needle shape pellets. Extrusion also has potential for microencapsulation of omega-3 oils [54].

## 5. Characterization of SLMs

The most important parameters in lipid particles characterization are particle size and size distribution, morphology analysis, drug loading, entrapment efficiency, and the release rate of bioactives.

### 5.1. Particle Size and Size Distribution

The size of the microparticles is a significant parameter in selecting application in foods, because it may cause undesirable sensory changes in products. It may also have a negative outcome on food texture and lead to product disqualification by the customers. Therefore, microparticle size should be <100 μm, not to harm food sensation [73]. The average size and size distribution can be evaluated by various methods, using appropriate instruments. Thus, the average hydrodynamic diameter of the Quercetin-loaded lipid microparticles produced from Babacu oil and denatured WPI was obtained by using photon correlation spectroscopy with a ZetaPlus instrument at 25 °C [29]. Particle size distribution as well as medium diameter (by volume) of solid lipid microparticles containing Vitamin B12 were determined by using a diffraction particle size analyzer (SALD 201 V) [50]. The same instrument was used for determination of particle size and size distribution of solid lipid microparticles loaded with Ascorbic acid (AA) [60]. To prevent clotting, microparticles were suspended in a Tween 80 aqueous solution (1% w/w) and stabilized for 5 min before analysis. Another useful method for the evaluation of microparticle size is laser diffraction. This technique was applied for particle size analysis of microparticles containing different concentrations of essential oils (EO) using Beckman LS 13320 instrument equipped with a universal liquid module and 2-propanol as dispersant [66]. Size distribution and mean volumetric diameter of the SLM encapsulating Ginger Oleoresin were determined by light scattering using laser diffraction in a Mastersizer 2000 [43]. A similar instrument, Mastersizer S, measured SLM size distribution of solid lipid particles containing Ascorbic acid [45]. An alternative method to measure solid lipid particle size and size distribution is evaluation by sieve analysis, using a vibrating shaker and four standard sieves of 100, 150, 250, and 500 μm [31].

### 5.2. Morphology Analysis

Morphological analysis of the obtained microparticles is usually performed by Scanning Electron Microscopy (SEM), enabling close visualization of the particle surface. Thus, Ascorbic acid (AA)-loaded solid lipid microparticles were visualized by SEM as spheres, characteristic for microparticles prepared by spray congealing method [59]. Scanning electron micrographs present more detailed analysis of the morphology and microstructure of SLM than optical microscopy. Using SEM technology, images of particles containing Vitamin-B12 revealed a smooth and continuous surface, typical for SLM obtained by the spray chilling method [50]. SEM images of solid lipid microparticles encapsulated Vitamin D3 revealed spherical particles with smooth surfaces that can promote the arrangement of microparticles, as well as powder flow and powder application [37]. Curcumin-loaded carnauba wax microparticles was also visualized by SEM (Shimadzu, SS55), revealing spherical morphology particles with diameters around 20 μm without fissures or surface irregularities [41]. SEM images of the microparticles prepared from vegetable fat made with partially hydrogenate cotton seed oil and loaded with soy protein hydrolysate (HP) exhibited spherical shapes and smooth surfaces, with varying diameters and agglomerated material [74].

### 5.3. Encapsulation Efficiency

There is no consensus in professional literature defining “encapsulation efficiency”, leading to different equations and methods, therefore making comparisons difficult. Generally, Encapsulation (Incorporation) Efficiency (EE) is defined as the ratio between the quantity of encapsulated material (core material) measured in SLM and the real value used to prepare the particles. The quantity of incorporated additives can be determined using HPLC or UV techniques. Usually, to measure this amount, solid microparticles should first be totally dissolved in an appropriate solvent, following by filtration, and evaluation by the chosen technique. Since a total amount of active ingredient is usually calculated in this way, to evaluate the actual encapsulated amount, a superficial amount should also be measured, then subtracted from the total amount. Efficiency of encapsulation is mostly dependent on interactions between the encapsulant (carrier, solid lipid) and encapsulated compounds (core, food additive), as well as on the encapsulation technique used [41]. The total encapsulation efficiency (TE) of Ascorbic acid (AA)-loaded SLM was determined by shaking the microparticles in chloroform, followed by the addition of distilled water [45,60]. After centrifugation, the concentration of AA in the aqueous phase was determined using the AOAC 967.21 method [75] (2,6-dichlorophenolindophenol titration method). The TE of the resulting microparticles was defined as the ratio between the total amount of ascorbic acid (AAT) in the sample and the initial amount of ascorbic acid added to the emulsion (AA0) (TE% = AAt/AA0*100). The superficial ascorbic acid (SAA) amount was determined by suspending the particles in a Polysorbate 80 solution. EE was calculated by subtracting the amount of SAA in the AAT in the sample. The ratio was expressed as a percentage representing the EE value: EE (%) = [(AAT-SAA)/AA0] × 100. Encapsulation Efficiency (EE) analyses of SLM containing Vitamin-B12 were evaluated by quantification of amount of Vitamin-B12 in the microparticles using HPLC method [50]. The EE was determined by similar equation: EE(%) = (TVC–CVF)/VCA) × 100%, where TVC is total Vitamin-B12, CVF is a superficial Vitamin-B12 and VCA corresponds to the amount of vitamins added during particles formation. The EE of solid particles bearing Gallic acid was also expressed as the difference between total and superficial gallic acid concentrations in relation to the amount of gallic acid added [62]. The gallic acid content of microparticles was quantified after particle dissolution in chloroform and extraction in the aqueous phase using the Foline-Ciocalteau colorimetric method, reading absorbance at 765 nm in a spectrophotometer. The amount of natural colorants Anthocyanin and Norbixin in spray-dried microcapsules was evaluated first by measuring the non-encapsulated color in the supernatant [57]. Then the pellet was dispersed in ethanol to quantify the total amount of color in the microcapsules, and the encapsulated color was measured using UV-Vis spectrophotometry. The color encapsulation efficiency (EE%) was calculated using the same equation. The actual Essential Oil (EO) amount in the microparticles was determined with gas chromatograph equipped with FID detector [66]. After determining the essential oil concentration in the microparticles and the theoretically expected essential oil concentration, the encapsulation efficiency was calculated using the following formula: EE% = ([EO in particles]/EO theoretical]) × 100.

### 5.4. Stability of Encapsulated Bioactives

Stability evaluation experiments are necessary in order to predict the behavior of the SLM and of active ingredient during extended storage. For example, the stability of the microencapsulated Ascorbic acid (AA) was evaluated on samples kept at 24 °C and 37 °C for 56 days [60]. Overall results showed that at least 70% of the initial AA was stable after 56 days. The stability of SLM loaded with proanthocyanidin-rich cinnamon extract (PRCE) was evaluated under storage conditions at 5 °C, 25 °C and 37 °C for up to 90 days [76]. Microparticles were evaluated for the content of proanthocyanidins, mean diameter, and crystal structure (XRD) during the storage period. The stability of Vitamin D3 incorporated into SLM was compared with free vitamin D3 during storage at 10 °C and 25 °C in closed vials in the dark for up to 65 days [37]. The results showed that SLM managed to preserve 71.8–86.3% of initial Vitamin D3 after 65 days of storage at room temperature, compared to 60.8% of free vitamin. In another study, the Anthocyanin and Norbixin content of the spray-dried microcapsules was evaluated at room temperature in the dark during one-month storage [57]. The natural color of microcapsules decreased sharply over time, as a result decomposition of natural colorants in contact with moisture present in produced powder at ambient temperatures. However, the encapsulation of Anthocyanin in microcapsules improved its stability against degradation compared to free Anthocyanin. In contrast, Norbixin-loaded microcapsules showed high retention without a significant reduction.

### 5.5. Gastrointestinal Stability of Encapsulated Phytochemicals and Food Additives

Generally, the nature of lipid system used in the formation of microparticles can be valuable not only for the improved intestinal absorption, but also for shielding the food active ingredients against acid and enzymatic degradation when they come in contact with gastric fluid (GF). In produced microparticles, the wall materials must tolerate gastric conditions, such as very low pH and enzyme pepsin existing in the stomach. However, upon approaching the small intestine, microparticles are expecting to release efficiently the core active materials [50]. Until now only few studies have extensively investigated the dissolution behavior of orally delivered SLM suitable for food applications. Therefore, only recently, one research concentrated on evaluating dissolution profiles of caffeine (CAF) from several formulations of SLM produced employing different lipids using various updated dissolution media to simulate conditions of the entire gastrointestinal tract (gastric tract and proximal human intestine) [31]. In these dissolution tests, CAF totally dissolved within 5 min both at pH 1.2 (stomach pH) and at pH 6.8 (small intestine pH), indicating high and pH-independent water solubility. It was found, that in the case of bigger particles (>250 μm), SLM composition was crucial for the dissolution behavior of active ingredients, while smaller SLM (100–250 μm) were mostly affected by the dissolution liquid content. A comprehensive review on factors affecting the bioavailability of β-carotene incorporated in solid lipid microcapsules was recently published [77]. The studies cited in this review show that both the simulated gastric medium and the nature of lipid microcapsules affect the bioavailability of β-carotene. A Vitamin-B12 release tests were performed as well on simulated gastric and intestinal system fluids [50]. SLM formed from vegetable fat and soy bean lecithin gradually released 90% of its encapsulated Vitamin-B12 in gastric fluid after 90 min of the experiment. When release studies were performed in simulated intestinal fluid, from the beginning of the analysis a high amount of vitamin was observed for all formulations. In another study, the survival of encapsulated probiotics was found to be above 75% when evaluated under simulated gastrointestinal conditions. The survival of probiotics was satisfied as well under different stress conditions (low pH, high level of sucrose and NaCl), showing that encapsulation improved their protection compared to the free form [36]. In the case of in vitro release behavior of caramel flavor, the release rate of the resultant particles in the simulated gastric environment was weak; however, the release rate in the simulated intestinal environment increased rapidly within 30 min. Approximately 70% of the core material was released within 120 min [56]. The taste-masking ability of microencapsulated powders was studied in vitro by dissolution of Quercetin-loaded SLM in the simulated salivary medium (pH 6.8). A significantly reduced dissolution rate of microencapsulated Quercetin in the simulated salivary medium in comparison to a non-encapsulated one may propose potentially good taste-masking effectiveness of this type of encapsulation [42].

## 6. Examples of Encapsulated Bio-Additives

### 6.1. Essential Oils (EOs)

Essential oils (EOs) are mixtures of natural volatile bioactive compounds produced by plants. Given their aroma, they are often used as flavors in the food industry. In addition, they have antibacterial, antifungal, and antiviral activities, also favoring their wide use as food preservatives in the food and feed industries [78,79]. Since synthetic chemical preservatives in food can be harmful to human health, EOs can offer better alternatives. However, the effective application of essential oils as natural preservatives and flavors in the food industry may be limited due to their volatile character as well as their reactivity and hydrophobicity. Thus, their incorporation into water-based foodstuffs requires encapsulation of EOs inside an appropriate matrix [80,81]. Using a microencapsulation technique, the storage stability of essential oils can be increased, the incorporation inside lipid particles can mask their unpleasant flavor, protect them from oxidation, and enable their controlled release (Table 5). The use of fats for encapsulation of essential oils can be highly favorable technique in light of their shared hydrophobicity. Thus, microparticles with different essential oil concentrations were produced by dispersing them within a hydrogenated vegetable fat matrix [66]. In another study, peppermint essential oil-loaded hollow solid lipid microparticles were successfully formed using a novel “green” method based on atomization of CO_2_-expanded lipid mixture from fully hydrogenated soybean oil (FHSO) functioning as a carrier [82,83,84,85,86,87].

### 6.2. Flavors

Flavors and fragrances are complicated chemical mixtures of volatile organic molecules with different physicochemical properties (e.g., volatility, water solubility) [71]. High consumer demand for the improved flavor of industrial food products led to encapsulation of flavor and aroma compounds. The primary objectives of encapsulation of flavors are to capture and retain the integrity of their complicated composition during the processing and prolonged storage. Flavors can be incorporated into mixtures using a wide variety of carriers. Encapsulated flavors are better protected against external oxidation yielding their greater stability. They also provide a dry version of what are usually liquid flavors, thus they can easily be worked into dry products [83].

#### 6.2.1. Coffee

Usually, consumers prefer their coffee based on its flavor. Coffee taste and smell involves several chemicals specific for the coffee bean roasting process. During prolonged storage, the coffee flavor can undergo several changes, amongst them loss of most volatile compounds, reactions between coffee components, and oxidation [56]. Temperature, oxygen, and moisture primarily affect the degradation reactions during these stages. Thus, there is a need to maintain coffee flavor for as long as possible to retain the quality of coffee beverages during its shelf-life. Microencapsulation of coffee flavor can protect flavor compounds from unwelcome chemical changes and may retain flavor mixture during industrial process or storage. A recent study concentrated on retention of the coffee flavor in a coffee-containing milk drinks by their microencapsulation using medium-chain triglyceride and maltodextrin as primary and secondary coating materials [56]. Authors have shown that microencapsulation of flavor and incorporation of these microparticles into coffee beverages improved quality due to effective flavor retention.

#### 6.2.2. Ginger Oleoresin (GO)

Ginger oleoresin (GO) is often used as a flavoring additive in foods. As a result of high viscosity and volatility of its natural liquid form, commercial Ginger oleoresin may cause some problems in handling during production. For example, some of its compounds may be decomposed or volatilized if exposed to heat and light during industrial processing or storage. Encapsulation techniques can be applied to reduce these difficulties, using food grade materials to protect and hold oleoresin. Thus, the spray chilling microencapsulation technique was used for the incorporation of GO into solid lipid microparticles (SLM) formed from mixtures of palmitic acid and oleic acid or palm fat serving as lipid matrixes [43,44].

### 6.3. Antioxidants

Antioxidants are frequently used in the food industry to prevent food decomposition through oxidation and to retain nutritional value and energy contents. They can also retain the freshness of products by protecting flavors, odors, color pigments, taste, and texture [59].

#### 6.3.1. Quercetin

Quercetin is a flavonoid that originated from a plant, which possess excellent antioxidant, anti-inflammatory, and anti-cancer properties [42]. However, due to its high lipophilicity, Quercetin has neglectable absorption in the gastrointestinal tract, leading to very low oral bioavailability: Less than 17% in rats and less than 1% in humans [29]. Besides its poor dissolution rate, it has a bitter taste that can pose a challenge for further development. Microencapsulation of Quercetin was proposed as a simple and attractive technique to produce taste-masked powders [42]. The obtained microparticles showed extended dissolution rates, indicating a potential for taste-masking. In other study, Quercetin-loaded solid lipid microparticles were prepared in order to protect Quercetin [29]. Results indicated that the obtained microparticles succeeded in protecting the incorporated Quercetin, retaining 85% of its starting amount after 30 days.

#### 6.3.2. Cinnamon

Cinnamon is a common spice used worldwide for culinary and medical purposes. Its extracts have many applications in foods and cosmetics due to their antioxidant, antimicrobial, anti-inflammatory, and antifungal properties. However, bio-actives can undergo fast deactivation or by binding to food components or by degradation by proteolytic enzymes when incorporated directly into food. To avoid stability problems, solid lipid microparticles loaded with proanthocyanidin-rich cinnamon extract (PRCE) were prepared using vegetable fat, a common raw material in the food industry [76]. In the above study, the formed PRCE-SLM successfully masked the unpleasant taste and astringent sensation of proanthocyanidins and other polyphenols from cinnamon extract.

#### 6.3.3. Guaraná

Guaraná is a native fruit of the Amazon rainforest. Guaraná seeds are rich in polyphenols, such as catechins and epicatechins that possess a high antioxidant activity. Recently it has been shown [84] that compounds found in commercial guaraná powder decrease oxidative stress markers in healthy subjects. As a result of their poor bioavailability and fast degradation under gastrointestinal conditions, it is difficult to preserve valuable properties of polyphenols [85]. The microencapsulation of guaraná seed extract (GSE) in solid lipid microparticles was achieved using a spray chilling method and vegetable fat as a carrier [40]. Microencapsulation was designed to protect the GSE during processing and storage as well as for controlling its release into the intestine. As a result of incorporation of guaraná seed extract in solid particles, its phenolic compounds remained relatively stable (at least 75% were detected after 90 days at 25 °C) and were released almost totally at simulated gastrointestinal media.

#### 6.3.4. Curcumin

Curcumin is a hydrophobic polyphenol obtained from the rhizomes of Curcuma longa. It has a strong yellow color and an extensive variety of beneficial biological properties, such as anti-inflammatory, anti-cancer, anti-microbial, and neuro-protective. Owing to these outstanding features and low toxicity, curcumin is an appreciated component used by the food industry as a natural yellow pigment instead of artificial colorants. Despite these features, application of this additive has been partial because of its high hydrophobicity, poor absorption, high sensitivity to light, and spicy flavor [71]. Encapsulation of the curcumin in solid lipid particles was proposed to overcome these drawbacks. In one study, Curcumin was incorporated into SLM formed from Babacu oil and Tristearin. The feasibility of producing low-sugar mango jams enriched with these curcumin-loaded lipid microparticles was examined [48]. Results indicated that the incorporation of SLM does not affect the consistency of prepared jams, and the obtained color was more intense and stable.

### 6.4. Natural Colorants

Due to increased demand for healthier food, natural colorants are widely used in foods and beverages instead of synthetic materials. In addition, natural pigments have been widely used for cosmetic and therapeutic purposes as well [86]. Most colorants are extracted from plant tissues that provide a wide range of colors and hues. Natural colorants are extracted from fruit and vegetable sources, such as saffron, berries, annatto, and beetroot [57]. Often, consumers prefer processed foods with not only attractive appearance and taste, but also that are healthy. This encourages food industries to search for new solutions and methodologies to meet consumer demand, safety requirements, and possess eco-friendly and biodegradable raw materials [87].

#### 6.4.1. β-Carotene

β-carotene (BC) is a red-orange pigment found in significant levels in various fruits and vegetables. It is considered to be a promising functional food ingredient and is used in food, cosmetic, and pharmaceutical applications as a natural pigment with nutraceutical values. However, the application of β-carotene is limited due to low water solubility and high chemical instability. Its high lipophilicity also decreases its bioavailability during digestion [39]. Solid lipid microparticles can be applied as transport systems to enhance the bioavailability of β-carotene as well as its protection. For example, solid lipid microparticle dispersions containing β-carotene and produced from palm stearin as the lipid phase have been combined with yogurts in an amount of 5% *w*/*w* (amount sufficient to provide suitable color) [77]. The presence of lipid microparticles did not change the physicochemical or the rheological characteristics of the produced beverage. In contrast, the addition of pure BC to the yogurts was not possible, since the hydrophobic nature of β-carotene prevented adequate mixing with the product.

#### 6.4.2. Lycopene

Lycopene is a natural pigment found widely in plants, which belongs to lipophilic isoprene family. It possesses varied bioactivity against cardiovascular diseases, eye diseases, nerval degenerative disease, and cancers. Lycopene is sensitive not only to possible industrial processing conditions, but also to oxidative degradation and cis-trans isomerization that can occur due to its 11 conjugated double bonds and multiple unsaturated olefin structures [88]. As a result of its low water-solubility and instability under gastrointestinal conditions, lycopene has low bioavailability, thus limiting its application in foods and health care products. The United Nations Food & Agriculture Organization, the Food Additives Committee, and the World Health Organization have recognized lycopene as a Class A nutrient. Developing effective methods to enhance the bioavailability of lycopene has become a popular research goal. Researchers are striving to develop different lycopene delivery systems [89] that can improve the absorption of lycopene in the gastrointestinal tract, enhance its chemical stability, and thus increase its bioavailability. One of these methods includes utilization of great solubility of lycopene in lipophilic solvents such as edible fats and oils and in the formation of solid lipid microparticles. In one study, solid lipid microparticles loaded with lycopene dissolved in sunflower oil solution have been produced from shortening, soy, and palm oils as the carrier [88]. In this way, it was possible to reduce lycopene degradation during 90 days of storage.

#### 6.4.3. Genipap

The genipap (*Genipa americana* L.) fruit is a source of blue colorants in Latin America with numerous possible applications [90]. Even though genipap has not been approved as a natural pigment in U.S. and E.U., the extract of genipap fruit permitted in the U.S. as a “Fruit Juice” color additive (21 Code of Federal Regulations, § 73.250). Like other natural colorants, genipap is sensitive material and prone to degradation under sever processing and storage conditions (e.g., light, heating, oxidizing agents, pH changes, etc.). In order to overcome these stability problems, Genipap-loaded microparticles were produced using stearic acid as a lipid carrier [90].

#### 6.4.4. Anthocyanin/Norbixin

Anthocyanin is a natural, water-soluble flavonoid extracted from the red and blue parts of plants such as red cabbage leaves, berries, and black carrots. It is one of the most frequently used pigments due to its exciting color. Norbixin is another water-soluble natural colorant extracted from the pericarp of Bixa orellana L. shrub seeds [57]. Both pigments have extensive applications in soft drinks, ice cream, yogurt, and yogurt drinks. Both Anthocyanin and Norbixin are highly subjected to thermal and photo-degradation, resulting in the formation of different effects and leading to a color change. Several methods have been developed to improve the stability of natural pigments in order to expand their potential applications in the food industry. Amongst these, solid lipid encapsulation has received much attention, since it can provide an effective physical barrier to protect natural colors from degradation. Solid lipid microparticles containing these heat-sensitive natural colorants were produced by assembling a core-shell structure with a shell including a glycerol mono-oleate (GMO) as a solid lipid shell, a water-dispersible polysaccharide layer, and an aqueous core housing [57].

### 6.5. Probiotics

Probiotics are live microorganisms that may improve public health by offering several benefits if consumed regularly in satisfactory amounts [36]. The demand for probiotic foods has increased as a result of consumer concerns about their food and health. The minimum recommended levels of probiotics in food have to be at least 106 cfu/g. Use of probiotic microorganisms in foods, however, has been partial due to the difficulty of preserving their viability during industrial processing and product shelf life. A recent study was initiated to evaluate the viability of Lactobacillus acidophilus (LA) and Bifidobacterium animalis subsp. lactis (BL) encapsulated in SLM using spray chilling, followed by adding them to savory cereal bars [67]. Encapsulated probiotics were more viable than lyophilized microorganisms, as expected, since the microorganisms inside the lipid carrier were protected from the presence of oxygen, water, and oxidative stress. Cereal bars with and without probiotics did not exhibit sensory differences, indicating that the inclusion of microorganisms encapsulated in solid lipid microparticles did not affect product sensory characteristics. The microencapsulation of probiotics provided a protective wall against the severe conditions of the gastrointestinal tract, such as low pH and the presence of enzymes and bile salts [36].

### 6.6. Fatty Acids

The growing demand for healthier products has caused the food industry to develop foods containing bioactives. Among these, omega-3 fatty acids, especially Eicosapentaenoic acid (EPA) and Docosahexaenoic acid (DHA), have attracted interest due to their unique health benefits [46]. The World Health Organization (WHO) and the North Atlantic Treaty Organization (NATO) recommend consumption of 0.3–0.5 g of EPA and DHA per day to obtain the benefits of EPA and DHA. Thus, there is a growing interest in food industry to develop foods supplemented with EPA and DHA. 

#### 6.6.1. Fish Oil

Fish oil is one of the main sources of EPA and DHA fatty acids essential for our central nervous system functions. Incorporation of fish oil into foods is challenging due to the low solubility of fish oil in aqueous food systems and its high susceptibility to oxidative deterioration during food processing and storage, thereby limiting their use in food products. Foods can be enriched with omega-3 rich oils using encapsulation techniques in order to improve consumption of omega-3 fatty acids. Microencapsulation of omega-3 oils can minimize oxidative decomposition, convert them from viscous liquid into easy-to-handle powder form, and extend shelf-life. Microencapsulation technology can offer practical solutions for the stabilization and improved delivery of omega-3 fatty acids in foods [46]. Recent study was concentrated on encapsulation of fish oil in hollow solid lipid particles formed from fully hydrogenated soybean oil (FHSO). These fish oil-loaded spherical solid lipid particles significantly improved the oxidative stability of the encapsulated fish oil compared to the free fish oil.

#### 6.6.2. Chia Oil

Chia (*Salvia hispanica* L.) oil has a high nutritional value, since it contains triglycerides with PUFA acids present in large proportions and an omega-3 content between 60% and 68%. PUFA are susceptible to oxidation that can lead to decreased nutritional and sensory quality. Chia oil was extracted and successfully encapsulated in stearic acid microparticles by hot homogenization method [51]. Researchers found that both omega-3 and omega-6 were effectively encapsulated, maintaining the same omega-3: omega-6 ratio present in natural oil. Microencapsulation enhanced the thermal stability of the chia oil as well.

### 6.7. Vitamins

‘Vitamins’ are defined as a group of micronutrients that cannot be synthesized by the human body. These compounds are classified into fat-soluble (A, D, E, and K) and water-soluble vitamins (B1, B2, B3, B5, B6, B7, B9, B12, and C) [91]. Vitamins play vital roles in our health due to their specific functions to regulate metabolic and cellular functions, promote health, reproduction and growth, and prevent disease. Vitamin deficiency can lead to severe diseases, such as scurvy, beriberi, and night blindness as well as neurological disorders. The stability of many vitamins is influenced by environmental impacts such as light, temperature, pH, humidity, and oxygen during food processing and storage. These factors usually lead to lose of the nutrient qualities of vitamins in final products, therefore encouraging food industries to develop new methods to prevent these losses by protecting vitamins in production and storage stages. Modern technology, such as microencapsulation, can offer the desired protection. One microencapsulation method is encapsulation in solid lipid particles. Examples of encapsulations of three different vitamins are presented below.

#### 6.7.1. Vitamin D

Vitamins D2 (ergocalciferol) and D3 (cholecalciferol) are the main forms of Vitamin D, a crucial nutrient for human health. Deficiency of Vitamin D in children is associated with rickets, while in the elderly the deficiency is the main cause of Oteomalacia [37,92]. Due to the connection between the possible skin cancer and exposure to sun radiation, the general population is facing insufficient levels of vitamin D in the blood, which can cause problems associated with Vitamin D deficiency. Supplementing and fortification are considered the best choices to fight Vitamin D deficit [93]. It is now common to consume supplements containing vitamin D, even though the actual available value of this vitamin is often limited by poor water solubility, rapid degradation, and low absorption, leading to low bioavailability. Researchers have concentrated on various techniques such as direct addition, emulsification, and microencapsulation to confront these problems. Since Vitamin D is a highly lipophilic compound, its incorporation into lipid particles is straightforward. In recent research, solid lipid microparticles loaded with 0.1% of vitamin D3 were produced by spray chilling method using vegetable fat as lipid carrier. In some formulations 1% beeswax and 1% soy lecithin were added as well in order to evaluate their influence on vitamin stability [37].

#### 6.7.2. Vitamin B12

Vitamin B12 is produced only by some organisms. This vitamin is highly essential for red blood cell production and central nervous system (CNS) functions. Since Vitamin B12 occurs only in few foods and in restricted quantities, a sufficient supplement of Vitamin B12 is extremely important. The U.S. Food & Drug Administration (FDA) determines the daily value for vitamin B12 to be at 2.4 mg. As Vitamin B12 is a highly sensitive compound, it can be easily decomposed when in contact with light, acid or basic environment, oxidizing agents, and prolonged heating [94]. Solid lipid microencapsulation offers an alternative to reduce difficulties associated with its instability. Vitamin B12-loaded SLM can be simply added to dark chocolate, peanut butter, or yogurts. It is known that the vegetarian or vegan population is mostly affected by the Vitamin B12 deficiency, therefore plant-derived foods can be considered as the best choice for lipid particles incorporation [50]. In a recent study, solid lipid microparticles loaded with Vitamin B12 were obtained by spray chilling technique using vegetable fat as the carrier. The results indicated that encapsulation provided increased protection of Vitamin B12 (>91.1% for all formulations after 120 days of storage) compared to the free vitamin (75.2%).

#### 6.7.3. Vitamin C (Ascorbic Acid)

Vitamin C, also known as ascorbic acid and ascorbate, originates from various foods and is sold as a common dietary supplement. Vitamin C is a crucial nutrient for the repair of tissues and the enzymatic formation of certain neurotransmitters. Vitamin C is essential for the functioning of several enzymes and is significant for the proper functioning of immune system. Ascorbic acid (AA) has been broadly used in the food industry for two different purposes—as an antioxidant additive, and as a source of Vitamin C in the diet [60]. Despite the efficacy of AA, like most of the other vitamins, it suffers from high chemical instability. Environmental influences, such as high pH, heat, oxygen, and UV rays, may affect its stability, thus weakening its nutrient value during the industrial production and prolonged storage of products. Ascorbic acid encapsulation may not only reduce its degradation, but it may also prevent contact with other components of the food matrix. In addition, it may also mask the special unpleasant acid taste of ascorbic acid. Several studies reported the use of spray chilling method for production of solid lipid microparticles containing ascorbic acid, using different lipids as wall materials, including fully hydrogenated palm oil and vegetable glycerol monostearate [60], Lauric acid (LA)/oleic acid (OA) mixtures [46], and stearic acid—hydrogenated vegetable fat mixtures [59].

## 7. Conclusions

Lipid based particulate systems have been used for the delivery of active agents in various fields, mostly for the delivery of pharmaceuticals. Entrapment of a drug in a lipid particle is intended to protect it from the environment, aqueous formulation or body fluids. Entrapment also allows a gradual exposure of the active agent to the delivery site or improve its physical properties towards crossing biological membranes. The use of lipid particulates in foods plays similar roles—protection and stabilization of actives, extended release, and improve activity. Lipid-based formulations use natural and edible ingredients that make them more accessible to foods than polymeric delivery technologies. With the increase interest in food quality and expansion of food technology, lipid particulate formulations are projected to grow exponentially.

## Figures and Tables

**Table 1:** Bioactive additives for health demand [1].

Anti-Ageing	Digestive Health	Immunity	Cognitive Health	Energy
Herbs/Botanicals	Probiotics	Herbs/Botanicals	Omega-3	Vitamins
Antioxidants	Herbs/Botanicals	Probiotics	Herbs/Botanicals	CoQ10
Omega-3	Enzymes	Antioxidants	Antioxidants	Herbs/Botanicals
Vitamin C	Fiber	Vitamin C	Ginger	Proteins
Vitamin E	Omega-3	Beta-sitosterol	Lecithin	Guarana

Business Insight Survey 2008, Market interest [2].

**Table 2:** Types of solid lipids.

Lipids	Chemical Composition	Properties *	Examples
Waxes	Esters of fatty acids and long chain alcohols	Hydrophobic mp = 62–86 °C	Carnauba wax, Candelilla wax, beeswax, solid paraffin, Rice Bran wax
Vegetable oils	Mixture of triglycerides, free fatty acids, phospholipids	Often digestible mp = 60–71 °C	Hydrogenated soybean oil, hydrogenated palm oil (Softisan 154)
Fatty acids	Long chain fatty acids	mp = 60–90 °C	Palmitic acid, Stearic acid, Behenic acid, Lauric acid
Triglycerides	Monoacid triglycerides	mp = 46–73 °C	Glyceryl tripalmitate (Dynasan 116), Glyceryl trimyristate, Glyceryl trilaurate
Fatty alcohol	Mixture of fatty alcohols	mp = 48–56 °C	Cetyl alcohol, Lauryl alcohol, Stearyl alcohol, Oleyl alcohol

* mp—melting point.

**Table 3:** Techniques for solid lipid microparticles production.

Method	Principles	Comments
Spray drying	Core material is dispersed into an aqueous encapsulant solution forming an emulsion or dispersion, followed by homogenization of the liquid, and then the atomization of the mixture into the drying chamber	The oldest and most commonly used method, flexible and lucrative process
Spray Chilling	Core material is dispersed into a coating solution and sprayed into a cold environment to solidify the carrier material	Suitable for temperature-sensitive cores
Extrusion	Emulsion dispersion containing core material passes through a die at high temperature and pressure into a bath for solidification of particles	Used primarily for encapsulation of flavors and other volatile cores in glassy matrices
High-shear homogenization	Dispersion of the additive-loaded lipid in hot aqueous surfactant solution, followed by high shear homogenization at a temperature above the melting point of lipid carrier and quick cooling	Cold homogenization for hydrophilic core material, hot for lipophilic core

**Table 4:** Comparison of particle properties obtained by different techniques.

Method	Size Distribution	Morphology	References
Spray drying	1.1 and 10.6 µm 10–50 µm—2–3 mm	Hollow, low density spherical particles with an irregular geometry	[57] [54]
Spray Chilling	10 µm 90–160 µm 200 µm	Dense particles with a spherical shape and smooth and continuous surface	[50] [60] [68]
Extrusion	0.3–5 mm 500–1000 µm	High density granules, sheets or strands can be obtained	[42] [54]
High-shear homogenization	1.09 µm 20 µm	Spherical particles with different density	[29] [41]

**Table 5:** Summary of encapsulated phytochemicals.

Food Additive	Examples	Reason for Encapsulation	Encapsulation Material	Ref.
**Antioxidants**	Cinnamon (proantho-cyanidins) Quercetin Curcumin Guarana	Low stability, unpleasant taste, rapid inactivation, degradation by proteolytic enzymes Low oral bioavailability, bitterness High hydrophobicity, poor absorption, low bioavailability, spicy flavor, high sensitivity to light Unstable in food processing and gastrointestinal conditions	Vegetable fat Babacu oil, Carnauba wax Babacu oil, Tristearin, Palm stearin Vegetable fat	[76] [29,42] [47,70] [40]
**Essential oils (EOs)**	Lemon, Peppermint, Carvacrol, Thymol	Storage stability, oxidation, controlled release, hydrophobicity	Hydrogenated vegetable fat Fully hydrogenated soybean oil	[66] [82]
**Fatty acids**	Fish oil (EPA & DHA) Chia oil	Low aqueous solubility, susceptibility to oxidation	Fully hydrogenated soybean oil Stearic acid	[46] [51]
**Flavors**	Coffee, Caramel Ginger oleoresin (GO)	Loss of volatile compounds, oxidation, degradation High viscosity, volatility, heat and light degradation	Medium-chain triglycerides (MCT) Palmitic acid with oleic acid or palm fat	[56] [43,44]
**Natural colorants**	Lycopene β-Carotene Genipap (*Genipa americana* L.) Anthocyanin, Norbixin	Oxidative degradation, cis-trans isomerization, low water-solubility, low bioavailability Low stability and bioavailability, high hydrophobicity, and sensitivity Thermal degradation, photodegradation Thermal and photodegradation	Hydrogenated and interesterified cottonseed, soy and palm oils Palm stearin Stearic acid Glycerol mono-oleate (GMO) and soy lecithin	[88,89] [39,77] [90,91] [57]
**Probiotics**	Lactobacillus acidophilus (LA), Bifidobacterium animalis subsp. lactis (BL)	Maintaining viability during processing and throughout the product’s shelf life	Vegetable fat	[36,67]
**Vitamins**	Ascorbic acid (Vitamin C) Vitamin D Vitamin B12	High instability by high temperature, high pH, and the presence of oxygen, metallic ions, UV and X-rays Poor water solubility, rapid degradation, and low absorption Degraded by light, heat, acid and basic media, and oxidizing agents	Lauric acid (LA)/oleic acid (OA) mixtures, vegetable glycerol monostearate, stearic acid—hydrogenated vegetable fat Vegetable fat Vegetable fat	[59,60] [37,92] [50]
